# Three-dimensional surgical simulation of the bile duct and vascular arrangement in pancreatoduodenectomy: A retrospective cohort study

**DOI:** 10.1016/j.amsu.2018.09.043

**Published:** 2018-10-16

**Authors:** Ryoichi Miyamoto, Yukio Oshiro, Naoki Sano, Satoshi Inagawa, Nobuhiro Ohkohchi

**Affiliations:** a Department of Gastroenterological Surgery, Tsukuba Medical Center Hospital, 1-3-1 Amakubo, Tsukuba, Ibaraki, 305-8558, Japan; b Department of Surgery, University of Tsukuba, 1-1-1 Tennodai, Tsukuba, Ibaraki, 305-8575, Japan

**Keywords:** 3D simulation, Hepatobiliary pancreatic surgery, Pancreas surgery, Pancreatoduodenectomy, Pancreatic cancer

## Abstract

**Background/aims:**

We evaluated the usefulness of three-dimensional (3D) images for pancreatoduodenectomy (PD), including the classification of the bile duct and vascular arrangement, i.e., hepatic artery, inferior mesenteric vein (IMV) and left gastric vein (LGV). We evaluated the extent to which this simulation affected the perioperative outcomes of PD.

**Methods:**

In all, 117 patients who underwent PD were divided into the without-3D (n = 53) and with-3D (n = 64) groups, and perioperative outcomes were compared. We evaluated the arrangement of the accessory bile duct and the hepatic artery (type I: the right hepatic artery arising from the superior mesenteric artery, type II: the left hepatic artery arising from the left gastric artery, type III: the most common pattern) and the confluence pattern of the LGV and the IMV [type i: portal vein (PV):splenic vein (SV), type ii: PV:superior mesenteric vein (SMV), type iii: SV:SV, and type iv: SV:SMV] between the two groups.

**Results:**

Two patients had an accessory bile duct. The 3D images were classified as type I (n = 4), type II (n = 10), type III (n = 48) and other patterns (n = 2); type ii (n = 27) was the most frequent confluence pattern (*p* < 0.05). Intraoperative blood loss was reduced in the with-3D group (*p* < 0.05).

**Conclusions:**

We propose that the 3D imaging technique is useful for preoperative assessment in PD.

## Highlights

• 3D simulation was particularly useful for understanding the bile duct anatomy.
• 3D simulation was assumed to lead to a reduction in intraoperative blood loss.
• 3D simulation was useful for preoperative assessment before pancreatoduodenectomy.

## 1 Introduction

Anatomical variations are frequently encountered in hepato-biliary-pancreatic surgeries, which necessitate a precise understanding of the positional relationships among the lesions, surrounding organs, and vessel arrangements to perform a safe surgery [1,2]. In particular, pancreatoduodenectomy (PD) requires detailed preoperative anatomical examination of the bile duct and vessels to prevent intraoperative injury [[3], [4], [5]]. Regarding the bile duct arrangement, previous studies have reported that the accessory bile duct, which is defined as an extrahepatic bile duct without a connection to the common hepatic duct within the liver, was present in 3.0–4.7% of patients undergoing bile duct surgery [6,7]. Moreover, Jonathan et al. and Koops et al. reported that unusual hepatic artery arrangements of the vascular components, i.e., the hepatic artery, portal vein (PV), left gastric vein (LGV) and inferior mesenteric vein (IMV), were present in 21–25% of patients undergoing hepatic surgery [8,9]. Sakaguchi et al. reported the confluence pattern of the LGV and IMV to the PV because it is known to have several anatomical variations [10].

Although recent advances in diagnostic imaging technology, such as multi-detector computed tomography (MDCT) and magnetic resonance cholangiopancreatography (MRCP), have enabled the collection of detailed information preoperatively, these methods have proven insufficient to determine the relative positions of the bile duct and vascular components, i.e., the hepatic artery, PV, LGV and IMV, and the parenchymal organs, such as the pancreas and liver. Therefore, we originally developed a method of merging MDCT and MRCP images and applied a 3D surgical simulation for pancreatic surgery [[11], [12], [13], [14], [15]]. By integrating these two image types, we have been able to understand the anatomical relationships between nearby vascular structures and the pancreas. Furthermore, we have been able to simulate the pancreatic dissection line and resulting anatomical image before performing the reconstruction procedure.

In the present study, we evaluated the usefulness of 3D surgical simulations for PD, including the classification of the bile duct arrangement and vascular components, i.e., the hepatic artery, PV, LGV and IMV. Furthermore, we evaluated the extent to which 3D surgical simulation affected the perioperative outcomes of PD.

## 2 Methods

### 2.1 Patient characteristics and perioperative outcomes

Between January 2011 and December 2017, we retrospectively analyzed 117 consecutive patients who underwent PD either with or without 3D surgical simulation (without-3D, n = 53; with-3D, n = 64). The ethics committee of the institution approved this study (H26-18). This study has been reported in line with the STROCSS criteria [16]. Since January 2013, we have routinely applied 3D surgical simulations to pancreatic surgery. Patient characteristics [e.g., age, sex ratio, body mass index (BMI), American Society of Anesthesiology (ASA) score, prior history of abdominal surgery, primary disease, and type of surgeon (surgical residents who were in their fourth to seventh postgraduate year or senior pancreatic surgeons)]; perioperative outcomes, including operating time, intraoperative blood loss, and length of postoperative hospital stay; and postoperative complications, including pancreatic fistula (PF), were compared between the two groups. Furthermore, we also evaluated PV resection, pancreatic texture and main pancreatic duct diameter as factors that influenced the perioperative outcomes [17,18]. We defined the pancreatic texture, i.e., hard or soft, and the main pancreatic duct diameter by the intraoperative findings or pathologic status of the pancreatic parenchyma. PF was defined according to the guidelines of the International Study Group on Pancreatic Fistula [19]. Postoperative complications excluding PF were graded according to the Clavien classification [20].

### 2.2 3D images used in the present study

**Fig. 1:**
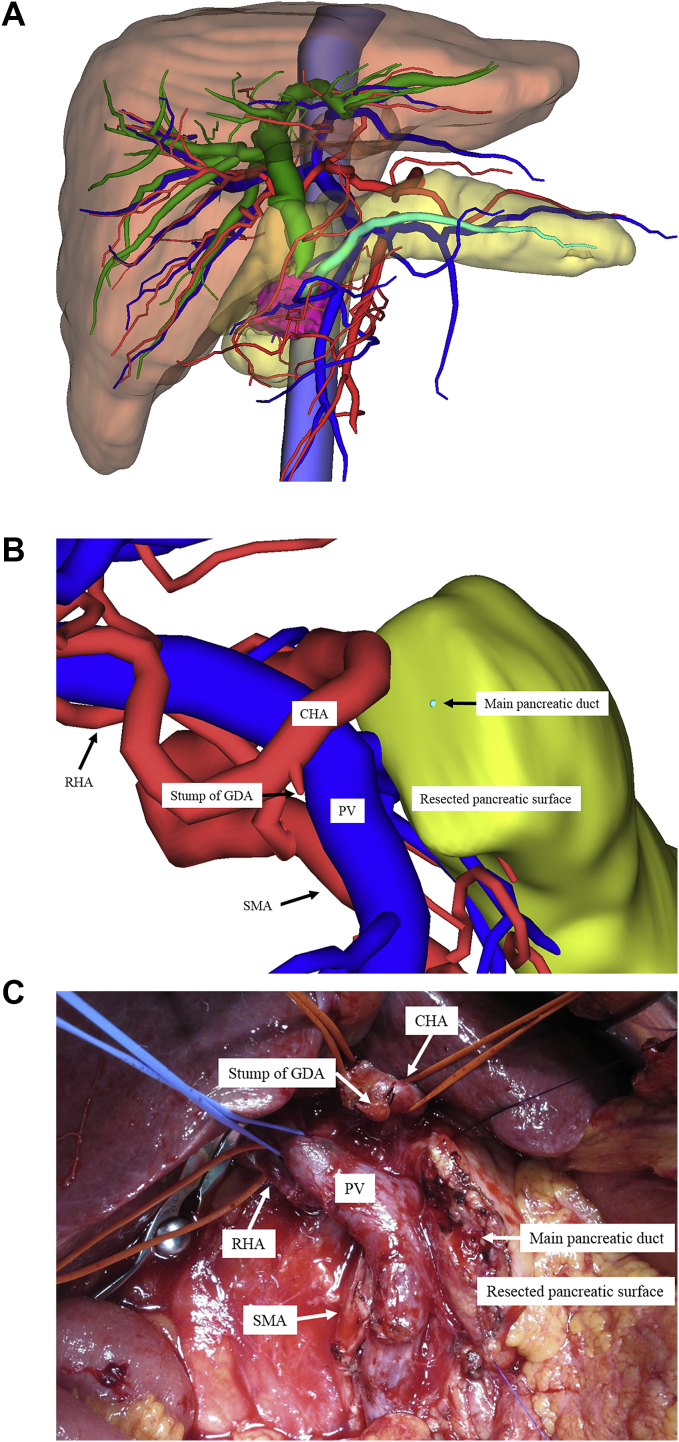
**A: Combined 3D image used in the present study.** A three-dimensional (3D) image from a pancreatic head cancer patient is shown. This view is an anterior 3D image. The red color represents the arteries; deep blue represents the veins, including the portal vein; light blue represents the inferior vena cava; green represents the biliary duct; turquoise represents the pancreatic duct; and pink represents the pancreatic tumor. **B: Simulated 3D image before performing the reconstruction procedure during pancreatoduodenectomy.** A simulated 3D image before performing the reconstruction procedure during pancreatoduodenectomy is shown. This view shows a patient's anterior right-side 3D image. The right hepatic artery (RHA) arising from the superior mesenteric artery (SMA). The red color represents the arteries; blue represents the veins, including the portal vein; turquoise represents the pancreatic duct. Abbreviations: CHA, common hepatic artery; GDA, gastroduodenal artery; PV, portal vein; RHA, right hepatic artery; SMA, superior mesenteric artery. **C: Intraoperative anatomical image before performing the reconstruction procedure during pancreatoduodenectomy.** An intraoperative finding before performing the reconstruction procedure during pancreatoduodenectomy is shown. This view shows a patient's anterior right-side 3D image. This figure was obtained from the same patient as in Fig. 1B. Abbreviations: CHA, common hepatic artery; GDA, gastroduodenal artery; PV, portal vein; RHA, right hepatic artery; SMA, superior mesenteric artery. (For interpretation of the references to color in this figure legend, the reader is referred to the Web version of this article.)

We used the SYNAPSE VINCENT^®^ medical imaging system (Fujifilm Medical Co., Ltd., Tokyo, Japan) to construct 3D images from MDCT images. We also obtained 3D images by integrating MDCT and MRCP images to produce accurate preoperative anatomical images (Fig. 1A). Furthermore, we were able to simulate the pancreatic dissection line and resulting anatomical images before performing the reconstruction procedure (Fig. 1B and C). By integrating these two image types, we could determine the anatomical relationships between the bile duct and the vascular components, i.e., the hepatic artery, PV, LGV and IMV, and the parenchymal organs, including the pancreas and liver. A preoperative conference also enabled the preoperative sharing of 3D anatomical images with the surgical staff [11,13].

### 2.3 Classification of the bile duct and vessel arrangements

**Fig. 2:**
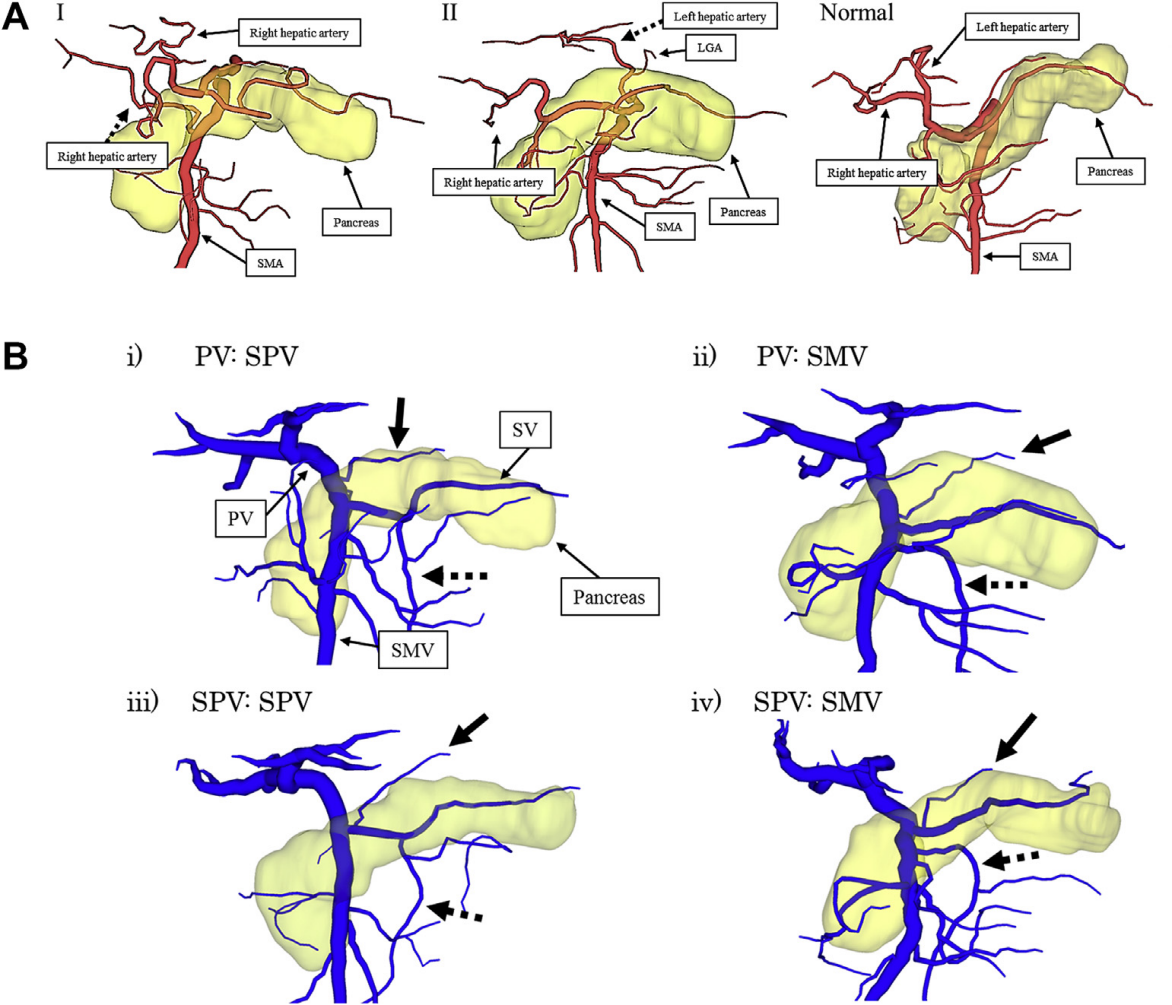
**A: Classification of the hepatic artery arrangement.** The course of the hepatic artery was divided into four groups [type I: the (accessory) right hepatic artery arising from the superior mesenteric artery (SMA) (black dashed arrow), type II: the (accessory) left hepatic artery arising from the left gastric artery (LGA) (black dashed arrow), normal type, and other patterns)]. **B: The confluence pattern of the left gastric vein (LGV) and the inferior mesenteric vein (IMV).** The confluence pattern of the LGV (black arrow) and the IMV (black dashed arrow) was divided into four groups [type i: PV:splenic vein (SV), type ii: PV:superior mesenteric vein (SMV), type iii: SV:SV and type iv: SV:SMV].

We evaluated the bile duct arrangement and vascular components, i.e., hepatic artery, PV, LGV and IMV, of 64 patients using 3D surgical simulations. We divided the course of the hepatic artery into four groups [type I: the (accessory) right hepatic artery arising from the superior mesenteric artery (SMA), type II: the (accessory) left hepatic artery arising from the left gastric artery (LGA), type III: the most common pattern, and other patterns] (Fig. 2A), and the confluence pattern of the LGV and the IMV was divided into four groups [type i: PV:splenic vein (SV), type ii: PV:superior mesenteric vein (SMV), type iii: SV:SV, and type iv: SV:SMV] (Fig. 2B). For the 53 patients without 3D surgical simulation, we also evaluated the bile duct arrangement and vascular components based on intraoperative findings.

### 2.4 Surgical procedures

All patients underwent subtotal stomach-preserving pancreaticoduodenectomy (SSpPD) and a modified Child's reconstruction [21,22]. A systemic regional lymphadenectomy was performed in all cancer patients; the lymph nodes included in the dissection were those in the hepatoduodenal ligament, the posterior pancreaticoduodenal nodes, and the nodes along the common hepatic artery. In cases performed with 3D surgical simulation, the surgical team observed the preoperative simulated 3D images on a large display during the surgery. Therefore, the surgical team could communicate and discuss the critical points of the surgical procedure. All surgical procedures were performed under the supervision of one or two senior pancreatic surgeons.

### 2.5 Statistical analyses

The correlations between the two groups were analyzed using the χ^2^ test or Fisher's exact test, as appropriate. Statistical analyses were performed using a statistical analysis software package (Version 21; IBM, Armonk, NY), and *p* values < 0.05 were considered statistically significant.

## 3 Results

### 3.1 Patient characteristics

The characteristics of the 117 patients who underwent SSpPD either with or without 3D surgical simulation (without-3D, n = 53; with-3D, n = 64) are presented in Table 1.

**Table 1:** Patient characteristics.

Factors	Without 3D (n = 53)	With 3D (n = 64)	*p value*
Age	69 (46–83)	66 (14–84)	0.310
Sex ratio (Male: Female)	36: 17	37: 27	0.112
BMI (kg/m^2^)	22.7 ± 2.15	20.8 ± 2.57	0.535
ASA score	2.02 ± 0.67	2.12 ± 0.55	0.756
History of abdominal surgery	8 (15%)	12 (23%)	0.118
Primary disease			
Pancreatic cancer	26 (49%)	32 (50%)	
Biliary cancer	15 (28%)	17 (27%)	
IPMN	8 (15%)	10 (16%)	0.156
Neuroendocrine tumor	2 (3.8%)	1 (1.5%)	
Others	2 (3.8%)	4 (6.3%)	
Operating surgeon			
Surgical resident	22 (42%)	20 (31%)	0.741
Senior surgeon	31 (58%)	44 (69%)	
Portal vein resection	9 (17%)	8 (13%)	0.117
Pancreas texture (Hard: Soft)	25: 28	33: 31	0.133
Main pancreatic duct diameter (mm)	3.51 ± 2.21	3.15 ± 1.98	0.552

Footnote: 3D, three-dimensional; BMI, body mass index; ASA, American Society of Anesthesiology; IPMN, intraductal papillary mucinous neoplasm.

**Table 2:** The bile duct and vessels arrangement.

Type of the bile duct and vessels arrangement	Without 3D (n = 53)	With 3D (n = 64)
Artery: the hepatic artery arrangement		
Type I	3 (5.6%)	4 (6.2%)
Type II	8 (15%)	10 (16%)
Normal	39 (74%)	48 (75%)
Others	3 (5.6%)	2 (3.1%)
Vein: the confluence pattern of the LGV and the IMV		
Type i	9 (17%)	13 (20%)
Type ii	24 (45%)*	27 (42%)*
Type iii	12 (23%)	14 (22%)
Type iv	8 (15%)	10 (16%)
Bile duct: the bile duct arrangement		
With Accessory bile duct	1 (1.8%)	2 (3.1%)
Without Accessory bile duct	52 (98%)	62 (97%)

Footnote: 3D, three-dimensional; LGV, left gastric vein; IMV, inferior mesenteric vein. *: p < 0.05 type ii versus other three groups.

No significant differences in the patient backgrounds were noted between the without-3D group and the with-3D group. The background characteristics compared included patient age, sex ratio, BMI, ASA score, prior history of abdominal surgery, primary disease, type of surgeon, PV resection, pancreatic texture, and main pancreatic duct diameter.

### 3.2 The bile duct and vessel arrangement (Table 2)

**Fig. 3:**
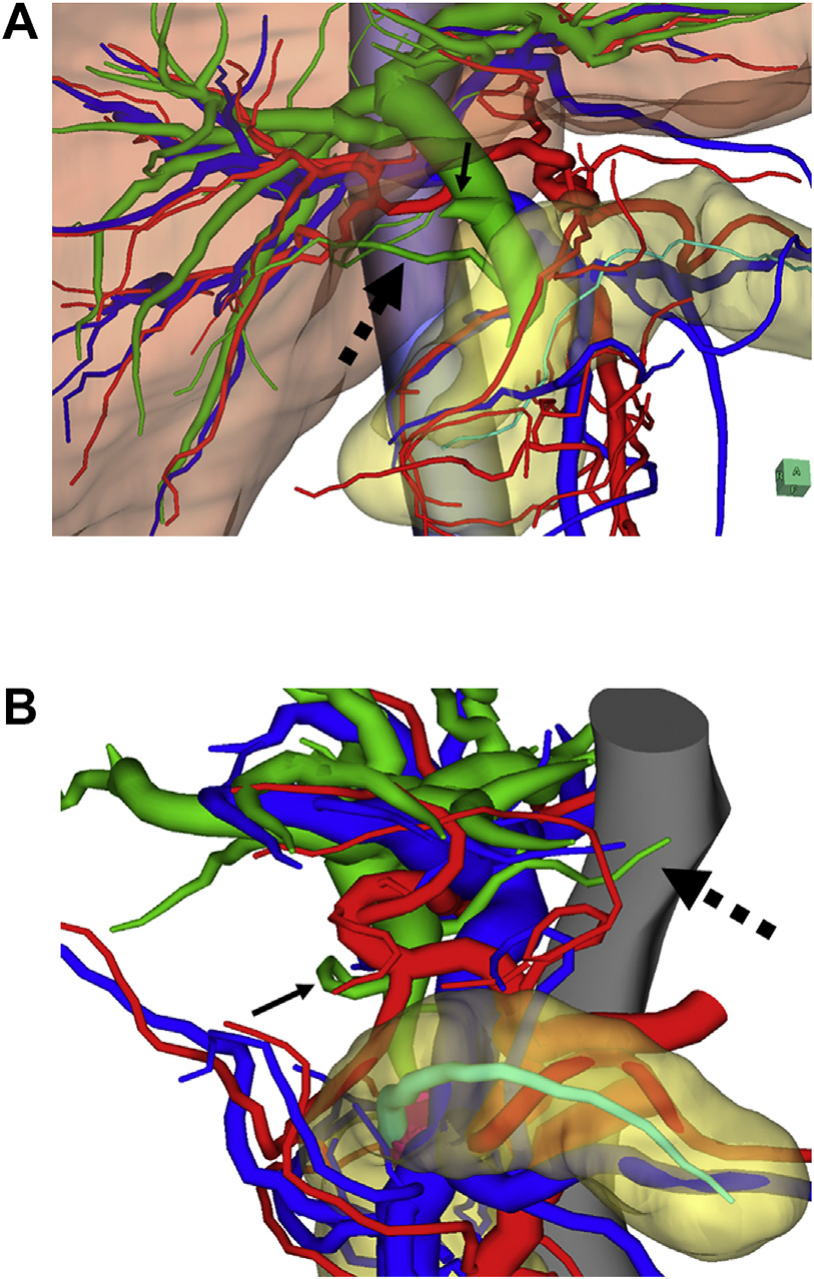
**Two cases with the accessory bile duct regarded as a caudate lobe bile duct. A:** This view shows a patient's inferior right-side 3D image. The connection of the accessory bile duct from the caudate lobe to the intrapancreatic bile duct (black dashed arrow) was easily recognizable. The cystic duct (black arrow) branched from the middle bile duct. **B:** This view shows a patient's superior left-side 3D image. The connection of the accessory bile duct from the caudate lobe to the middle bile duct (black dashed arrow) was easily recognizable. The cystic duct (black arrow) branched from the middle bile duct.

In terms of the bile duct arrangement on 3D imaging, two patients exhibited an accessory bile duct, which was regarded as a caudate lobe bile duct. One patient had a caudate lobe bile duct that was connected to the inferior common bile duct (Fig. 3A). The other patient had a caudate lobe bile duct that was connected to the middle common bile duct (Fig. 3B). One patient among the patients without 3D surgical simulation also exhibited an accessory bile duct, which was regarded as a caudate lobe bile duct.

Regarding the hepatic artery arrangement on 3D imaging, 4 patients (6.2%) had type I, 10 patients (16%) had type II, 48 patients (75%) had the normal type, and 2 patients (3.1%) were classified as other patterns. Among the patients without 3D surgical simulation, 3 patients (5.6%) had type I, 8 (15%) had type II, 39 (74%) had the normal type, and 3 patients (5.6%) were classified as other patterns.

Regarding the confluence type of the LGV and IMV, 13 patients (20%) had type i, 27 patients (42%) had type ii, 14 patients (22%) had type iii, and 10 patients (16%) had type iv. The type ii confluence pattern was the most frequent pattern among these four groups (*p* < 0.05). For the patients without 3D surgical simulation, 9 patients (17%) had type i, 24 (45%) had type ii, 12 (23%) had type iii, and 8 patients (15%) had type iv. The type ii confluence pattern was also the most frequent pattern among these four types (*p* < 0.05) among this group of patients.

### 3.3 Perioperative outcomes

A significant difference was observed for the intraoperative blood loss, which was 1174 g in the without-3D group and 810 g in the with-3D group (*p* = 0.012). However, a comparison of the perioperative outcomes between the two groups did not reveal significant differences in the operating time, length of postoperative hospital stay, incidence of Grade III-V complications, or incidence of Grades B and C PF (Table 3).

**Table 3:** Comparison of perioperative outcomes between the two groups.

Factors	Without 3D (n = 53)	With 3D (n = 64)	*p value*
Operating time (minutes)	498 ± 169	451 ± 153	0.453
Intraoperative blood loss (g)	1174 ± 862	810 ± 668	0.012*
Postoperative complications (Clavien's classification)			
Grade I, II	2 (3.8%)	9 (14%)	0.411
Grade III, IV, V	2 (3.8%)	4 (6.3%)	0.239
Pancreatic fistula (ISGPF classification)			
Grade A	12 (23%)	8 (13%)	0.132
Grade B, C	11 (21%)	19 (29%)	0.252
Length of postoperative hospital stay (days)	15 (8–51)	20 (10–160)	0.117

Footnote: 3D, three-dimensional; ISGPF, International Study Group on Pancreatic Fistula. *: p < 0.05 with 3D versus without 3D.

## 4 Discussion

Our 3D images, created by integrating MDCT and MRCP images, could produce accurate preoperative anatomical images and enabled us to determine the relative positions of the bile duct, vascular components and parenchymal organs during PD. Furthermore, vascular arrangements including the hepatic artery, PV, LGV and IMV were definitively classified by the 3D images.

Healey defined the accessory bile duct as an extrahepatic bile duct without a connection with the common hepatic duct within the liver [23]. Miyakawa et al. reported an accessory bile duct in 21 of 450 (4.7%) patients [6]. Similarly, Hisatsugu et al. reported an accessory bile duct in 616 of 19,892 (3.1%) patients undergoing bile duct surgery [7]. They classified the accessory bile duct into seven categories based on the position of the common bile duct. In the present study, 2 patients (3.2%) had an accessory bile duct. According to Hisatsugu's classification, the present accessory bile ducts connected to the inferior and middle common bile duct were classified as type IV and type III and comprised 3.0% and 57.4% of the total patients, respectively [7].

Considering that major bleeding occurs primarily from laceration of the fragile veins, the precise recognition of the LGV and IMV anatomy is of considerable importance. Sakaguchi et al., utilizing 3DCT portography, previously reported that the most frequent confluence locations of the LGV and IMV were the SV [10]. By employing 3D imaging, the present study clearly classified the confluence pattern of the LGV and the IMV around the head of the pancreas. Furthermore, we found that the most frequent confluence pattern of the LGV and the IMV was the PV and SMV type.

When systemic regional lymphadenectomy in the hepatoduodenal ligament is performed, precise recognition of the hepatic artery arrangement is required. According to our classification, Hiatt et al. reported a type I pattern in 106 (10%), type II pattern in 97 (9.7%), a normal type in 757 (75%) and other patterns in 38 (3.8%) of 1000 patients with donor livers that were used for orthotopic transplantation [9]. Using angiography, Koops et al. similarly reported a type I pattern in 72 (12%), type II pattern in 26 (4.3%), normal type in 477 (79%) and other patterns in 29 (4.8%) of 604 patients [8]. Regarding our classification, our results were assumed to be similar to those of previous reports. Because we did not use angiography, we assumed that our reconstructed 3D imaging technique was less invasive for patients.

In the present study, we observed a reduction in intraoperative blood loss in the with-3D group compared to that in the without-3D group. This result suggested that preoperative understanding of the 3D anatomic imaging, especially for the arrangement of the bile duct, hepatic artery, PV, LGV and IMV, and sharing of the anatomical images with the surgical staff contributed to the reduced intraoperative blood loss in conjunction with the mastery of surgical techniques, advances in surgical instruments and perioperative management. Our previous study indicated that the preoperative 3D reconstruction during liver surgery significantly reduced the operating time. Therefore, we proposed using the preoperative 3D reconstruction technique during hepato-biliary-pancreatic surgeries, which present various anatomical variations, as a useful modality for preoperative assessment and intraoperative navigation to perform a safe surgery [24].

Recent developments in MDCT radiological technology, which provide rapidly acquired multiphase thin datasets in the early/delayed phase, allow for the continuous acquisition of MDCT images. In 2001, Kamel initially reported the reconstruction of 3D images for surgical planning in potential donors, which was evaluated for live adult right lobe liver transplantation [25]. Many institutes in Japan began to construct 3D images from MDCT datasets of patients undergoing hepatic resection to facilitate surgical planning and to allow for the sharing of complicated anatomical images with the surgical staff [26,27]. In hepato-biliary-pancreatic surgeries, information regarding the bile duct arrangement is essential, and the 3D simulations from MDCT imaging alone appear to be insufficient. We originally developed 3D images by integrating MDCT and MRCP images to produce accurate preoperative anatomical images, and we applied this method to hepato-biliary-pancreatic surgery, including PD [11,[13], [14], [15]]. By integrating these two imaging techniques, it is possible to better understand the anatomic relationships among the bile duct arrangements, vascular components, i.e., hepatic artery and PV, and the parenchymal organs, i.e., the pancreas and liver. Moreover, preoperative sharing of the anatomical 3D images with the surgical staff becomes possible. The present study has limitations, as it was a study of a relatively small number of patients. Hence, these results will need to be prospectively confirmed by additional multi-institutional, large-scale studies.

## 5 Conclusion

The present 3D surgical simulation technique was useful for understanding and sharing relative anatomic information concerning the bile duct and vascular components, which is essential for safe PD. We propose the use of this 3D imaging technique as a new practice for preoperative assessment and intraoperative navigation when performing PD.

## Ethical approval

The ethics committee of the University of Tsukuba Hospital approved this study (H26-18).

## Sources of funding

The authors have no funding for this reasearch.

## Author contribution

Protocol/project development: All authors.

Data collection or management: Ryoichi Miyamoto.

Data analysis: Ryoichi Miyamoto.

Manuscript writing/editing: Ryoichi Miyamoto and Yukio Oshiro.

## Conflicts of interest and source of funding

The authors have no conflicts of interest or financial ties to disclose.

## Research registration number

This study was registered in Researchregistry4284.

## Guarantor

Ryoichi Miyamoto.

## Provenance and peer review

Not commissioned, externally peer reviewed.

## Appendix A Supplementary data

the_strocss_statement_checklist the_strocss_statement_checklist
